# Ecological drivers of female lion (*Panthera leo*) reproduction in the Kruger National Park

**DOI:** 10.1002/ece3.5935

**Published:** 2020-04-15

**Authors:** Nkabeng T. Maruping‐Mzileni, Sam Ferreira, Kim Young, Paul J. Funston

**Affiliations:** ^1^ Scientific Services South African National Parks Kimberley South Africa; ^2^ Scientific Services South African National Parks Skukuza South Africa; ^3^ Cheetah Program Panthera NY USA; ^4^ Lion Program Panthera NY USA

**Keywords:** conception, Kruger National Park, lion, panther leo, reproduction

## Abstract

The role of social cues in the reproduction of social mammals, particularly carnivores, has been thoroughly studied and documented in literature. However, environmental cues such as resources of water, food, and shelter have been identified to a lesser extent. Pregnant lions (*Panthera leo*) are notoriously secretive during the final stages of pregnancy and postpartum. Behavioral indicators depicted by movement patterns obtained by remote detection of collared female lions in the Kruger National Park were necessary for the monitoring of birth timing. Over the study period, eight plus a potential three parturition incidences of collared females were recorded. Of the variables measured (step length, range size, duration, prey biomass, and rainfall), range size during the month of parturition was the most indicative movement pattern of a successful birth. By backdating the potential birth month of the litters, date of conception was calculated and our results revealed a correlation between the birthing peaks of preferred prey during the month of conception. Birth timing in conjunction with remote sensing and ecological factors were thus identified behaviors associated with denning.

## Graphical abstract

Behavioural indicators depicted by movement patterns obtained by remote detection of collared female lions in the Kruger National Park can reduce the search area for reproductive lions. The information informs management on activities such as landscapes to include in protected areas, avoiding fire or other disturbing activities.

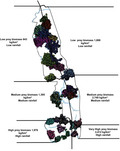

## 1 INTRODUCTION

Reproduction of social mammalian species relies on ecological, environmental, and social cues (Bertram, 1975). Social cues such as changes in dominance or groups dynamics are equally important for large carnivores and have been found to influence the reproductive activity of social species such as spotted hyenas, wild dogs (*Lycaon pictus*), and red wolves (*Canis rufus*; Creel, Creel, Mills, & Monfort, 1996; Hinton & Chamberlain, 2010; Holekamp, Szykman, Boydston, & Smale, 1999). Some benefits of social reproductive cues are synchronized births among group members resulting in better survival of young, typically complemented by cooperative rearing and protection (Dellinger, Ortman, Steury, Bohling, & Waits, 2011; Pusey & Packer, 1994).

Ecological and environment cues in the form of rainfall and prey biomass have been documented to strongly contribute to reproductive activity of large carnivores (Holekamp et al., 1999). For example, spotted hyenas (*Crucuta crocuta*) in the Maasai Mara National Park, Kenya, have synchronized birth peaks coinciding with peaks of food abundance. A similar pattern was recorded for lions (*Panthera leo*) in Nairobi National Park, Kenya (Holekamp et al., 1999; Rudnai, 1973). Both these studies, however, only focused on birthing incidences and ecological conditions. Although ecological and environmental cues such as rainfall and prey biomass are identified as factors influencing reproductive schedules of lions, research into these cues is limited (Bertram, 1975; Ogutu & Dublin, 2002).

Rainfall influences resource availability, such as food, water, and shelter, which are critical for the successful rearing of cubs (Packer et al., 2005; Van Orsdol, Hanby, & Bygott, 1985). The overriding driver that influences interactions and survivorship of African predators is resource availability, mainly rainfall (Maruping‐Mzileni, Funston, & Ferreira, 2017). Seasonality of rainfall determines the amount of available surface water; this in turn influences the distribution and abundance of lion prey species. This is because energy is required for gestation, lactation, and eventual protection of offspring. Changes in energy requirements suggest an alteration of physiological demands that manifest in behavioral changes. Literature suggests that a behavioral change independent of environmental changes indicated by altered or reduced movement patterns could indicate potential physiological stress (Hinton & Chamberlain, 2010) such as pregnancy.

The spatial ecology of lions is based on their need to fulfill physiological, ecological, and social requirements (Ogutu & Dublin, 2004). These requirements are met based on habitat suitability (Ogutu & Dublin, 2002), resource availability (Packer et al., 2005), and social dynamics (Loveridge et al., 2009). Home ranges are large enough to ensure access to key resources such as food, water, and shelter and in some cases are defended either fully or in part as territories. Lions will adjust their location in space until the requirements have been met and in so doing define a home range (Beyer et al., 2012).

Prey movement and abundance are significant determinants of home range size and use by lions (Loveridge et al., 2009; Morales et al., 2012). Lion home ranges are smaller in areas of high prey availability compared with those of lions living in areas of low prey availability (Packer et al., 2005). De Boer et al. (2010) found that animal distribution is nonrandom and tend to congregate around water where lion hunts occur most frequently. These findings suggest that resource availability in the form of prey and water are key drivers of lion home range dynamics. Consequently, home range dynamics may thus influence reproductive activity of lions.

The ability to survive and access all necessary resources can be compromised by physiological stress. Disease, injury, chronic food restriction, or pregnancy can impose physical stressors on the body such that limit an individual's ability to obtain resources. For example, these stressors have been found to exacerbate and influence behavioral patterns and movement patterns (Wu, Bazer, Cudd, Meininger, & Spencer, 2004). Chronic food restriction experienced or injuries are clear examples of physiological stress. Physical stress resulting from chronic food restriction altered reproductive development and estrous cycles of spotted hyenas and lions in the Maasai Mara National Park (Holekamp et al., 1999) lions in Nairobi National Park (Rudnai, 1973) and sheep in New Zealand (Rae et al., 2002). Similarly, when seasonal rainfall and ecological conditions favored the success and thus an increase in wildebeest numbers of the Serengeti, lion cub survival increased because of an increase in resource availability (Packer et al., 2005). Disease and pregnancy, however, are less understood as physical stressors that limit the ability to access resources. Bovine tuberculosis (bTB) is an emergent infectious chronic disease that was opportunistically identified in buffalo (*Syncerus caffer*) of the Kruger National Park (KNP) in the 1960s and in lions in the 1990s (Renwick, White, & Bengis, 2007). Symptoms include swollen joints, weight loss, and lameness (Renwick et al., 2007). Such physical stress can reduce a lion's ability to obtain resources, influence social interactions, and reduce energetic expenditure for reproduction.

Here, we hypothesized that for adult female lions, energetic constraints such as the ability to hunt or obtain resources are imposed when the body is stressed during pregnancy as a result of factors such as disease. The inability to access resources can potentially result in reduced conception rates, reduced ability to maintain pregnancy, and changes in movement patterns to establish a denning site where cubs are born and reared. Therefore, we asked (a) do lions show seasonality of reproduction, (b) is birth timing a function of ecological factors, and (c) is fecundity a function of ecological factors?

To test these hypotheses, we first determined the potential month when a birth occurred through several indicator variables collected through GPS radio‐telemetry and GPS satellite. The indicator variables measured were the number of fixes at a localized site, the elapsed time of consecutive fixes at a localized site, and the distance between the fixes. We then verified whether a successful birth occurred according to the following: (a) the duration of stay at a particular denning site using GPS fix clusters; (b) core and total home range size during the potential birth month, and then (c) variation in daily net displacement (km) during the wet and dry seasons; and (d) variation in daily net displacement (km) during the potential birth month. These variables were chosen because they are the best indicators of changes in prenatal and postnatal behavioral patterns. To test for the season of reproduction, we measured movement patterns by calculating net displacement as a function of energy expenditure and range size as a function of resource availability. To test for the association of ecological factors on birth timing, we calculated prey biomass and rainfall as a function of resource availability. To test the association of fecundity with ecological factors, we compared the KNP with other game reserves as benchmarks.

## 2 METHODS

The KNP is situated in South Africa's eastern low‐lying savannah (between 22°25′–25°32′ South and 30°50′–32°02′ East) and comprises woodlands (*Sclerocarya birrea* and *Acacia nigrescens*), shrubveld (*Colophospermum mopane*), thickets (*Acacia* spp. and *Combretum* spp.), plains (*Themeda triandra* and *Bothriochloa radicans*), and bushveld (Mucina and Rutherford, 2006).

**Figure 1:**
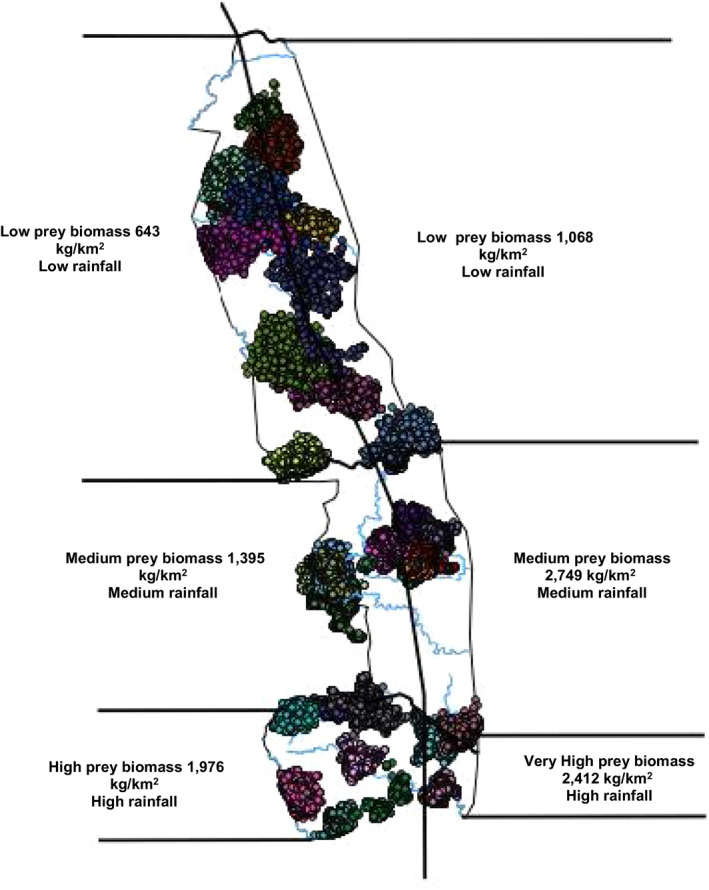
Ecological zones of Kruger National Park according to available prey biomass and the home ranges of lions collared between 2010 and 2012 (Ferreira & Funston, 2010)

Mean annual rainfall varies from the north to the south between 450 to 750 mm per annum, respectively. Precipitation generally decreases from south to north and increases from east to west. The wet season is from October to March and receives approximately 80% of the precipitation, with the driest months being April to August (Du Toit, Biggs, & Rogers, 2003). Relative temperature ranges from 0 to 40°C, with high peaks from December to February. June and July are the coldest months when frost occurs, particularly in the low‐lying areas. We divided KNP into three zones according to prey abundance, and rainfall, with all factors being highest in the south and lowest in the north. Given the vastness of the KNP landscape, to best classify the environmental and ecological factors, we used Acocks (1988) vegetation classification. The ecological factors used to classify the study area into regions were prey biomass and average rainfall. The regional categories were the southern region which had high prey biomass, and high rainfall and the northern region which had low prey biomass, and low rainfall (Figure 1; Ferreira & Funston, 2010).

This study formed part of a registered KNP and South African National Parks (SANParks) Scientific Services project and as such followed strict ethical standards for capture and handling of lions (SANParks Standard Operating Procedures). All permits necessary to conduct the research in the KNP were obtained. Ethics committee approvals were obtained from both Tshwane University of Technology and SANParks. All capture work and veterinary work were conducted under the supervision of the SANParks Veterinary Wildlife Services (VWS). All persons assisting during captures held required expertise and met levels of efficiency for processing captured individuals and collecting samples. Data will be stored on the SANParks Data Repository, metadata is available from http://dataknp.sanparks.org/sanparks/metacat/judithk.112037.1/sanparks, and full data made accessible on request.

We lured and captured the lions using the mass‐capture standard call‐up station method (Ferreira, Maruping, Schoultz, & Smit, 2013) between 2010 and 2012. At each capture event, a caravan was parked 20 m from the bait and a light delivery vehicle with an open load bed was used as recovery vehicle after lions were immobilized. Veterinarians used formalized drug combinations (SANParks, unpublished data) according to an individual's age and sex for darting. Lions were darted using a combination of medetomidine and zoletil (a combination of tiletamine and zolazepam) delivered from the caravan or vehicle with a Dan‐Inject dart gun. Lions were darted and then processed. Processing involved a restricted team of four skilled people retrieving an animal from the bait and transferring it to the processing site within the protective vehicle circle using the light delivery vehicle (Ferreira et al., 2013). Once immobilized, assessment of captured lions by veterinarians from the Veterinary Wildlife Services (SANParks‐VWS) included general health and body condition such as muscle tone, coat condition, dehydration and reproductive state, age assessment, and tissue sampling for assessing disease status. During subsequent monitoring, demographic information including total pride size and structure was identified and recorded.

In each pride captured, one adult female was fitted with a collar. In the southern region, where prey biomass and rainfall is high, captures (*N* = 10) took place between February and March 2010 while in the northern region, where prey biomass and rainfall is low, captures (*N* = 13) were conducted from August to September 2010. We also captured lions throughout the study period when collars needed to be changed. Of the collared females in the northern region, only 10 were used for this analysis because of inconsistent data when collars failed or females were not seen for more than three months.

An adult female from each of the pride was fitted with a standard mammalian collar. The collar includes a battery pack and a built‐in tracking unit and antennae sewn between the belt layers. The width and weight is species‐specific (www.awt.co.za) [Accessed: 2015, 22 July]. All collars had Global Positioning System (GPS) and Very High Frequency (VHF) radio capability. Prides in the southern region had collars with cellular Global System for Mobile (GSM) GPS download while the prides in the northern region had satellite GPS download. Body condition was scored 1–5 according to the Packer and Whitman (2005) scale of all adult females to reflect the reproductive and physical condition. According to the Packer and Whitman (2005) scale, body condition of 1 is emaciated and 5 is obese.

Behavioral indicators such as range use, diet and movement patterns have been used by Packer, Pusey, and Eberly (2001) to identify stress and energetic constraints. The integration of GPS technology and direct observations allowed improved overall data quality to observe behavioral indicators (Tomkiewicz, Fuller, Kie, & Bates, 2012). The upload and fix frequencies for each collar were set at four‐hour intervals. GSM‐GPS collar data were uploaded and accessed from the African Wildlife Tracking website (www.awt.co.za) [Accessed: 2010, 31 June] when the animal was within cellular network range. Satellite GPS collars uploaded fixes every 4 hr onto the MS Track (http://mstrackweb8.skygistics.com) and two African Wildlife Tracking websites: (www.awt.co.za) and (http://www.awt.awetelemetry.com). Variations in vegetation structure, terrain, climatic factors, and power lines may have affected collar upload frequency, accuracy, and precision (Di Orio, Calla, & Schaefer, 2003). Of the total number of fixes obtained, the fix percentage was approximately 90% (*SD* ± 20). Satellite collars were the most reliable with low failure‐to‐download incidences.

Duration at a site was calculated by measuring the amount of time spent at a denning site or at a locality. The duration of stay was calculated by identifying clusters defined as the number of consecutive fixes within a 200 m radius. The information included longitude, latitude, altitude, temperature, date, and time. We used the data from the GPS fixes to measure daily movement patterns of the collared lionesses.

### 2.1 Data analysis

For each collared female in the north and south, we used generalized linear models (GLMs) to identify which of the explanatory variables (range size, daily net displacement, and rainfall) were most indicative of the likelihood of a birth. These models do not force data into predefined artificial scales, thus allowing for nonlinearity and nonconstant variance structures in the data (Hastie & Tibshirani, 1990). We compared Akaike information criterion (AIC) among candidate models with a single and combination explanatory variables (Johnson & Omland, 2004) for the southern and northern region. From the AIC, corrected AIC*c* was calculated. This adjusts the AIC for small sample sizes. The AIC*∆* was then calculated, which is the difference between the AIC value of all candidate models and the candidate model with the lowest AIC score. The Akaike weight (*w*j) was then derived from the candidate model averages to determine the relative likelihood of the model (Johnson & Omland, 2004). This method was used because it independently evaluates the model discriminatory power (Wisz et al., 2008). A candidate model for the duration of stay was not run due to the subjective nature of the data.

Range size (core 50% and total 90%) and daily net displacement were measured in ArcGIS 9^®^ ArcMap v9.3 (Environmental Systems Research Institute, Midrand, Gauteng, South Africa) using LoCoh (Getz et al., 2007) and Animal Movements found in Hawth's Tools (http://www.spatialecology.com/htools) [Accessed: 2012, 22 March]. Net displacement was calculated as the distance (km) between consecutive GPS fixes. This was then transformed into a daily net displacement by taking the average distance between fixes in a 24‐hr period. The schedule and frequency of GPS fixes for all collars were uploaded every four hours and commenced in February of 2010 in the southern region and in August 2010 in the northern region until December 2012. When collars failed, only GPS fixes from observations were recorded for movement purposes. The data, however, could not be used for calculating net displacement. Of the total fixes collected, the cellular GSM‐GPS collars had a 15% failure rate and the satellite GPS collars had a 10% failure rate over the three year study period.

Duration of stay at denning sites was calculated as the number of consecutive days spent within a 200 m radius. The radius of 200 m was chosen because this indicates localized movement and displacement. An excess of 200 m would suggest relocation away from an area (Tambling, Cameron, Toit, & Getz, 2010). Fixes were taken at four‐hour intervals with time associated with when the fix was uploaded and could thus be calculated into days spent at a location. The southern and northern regions were analyzed separately and then compared to distinguish the differences in birth timing (season of occurrence), fecundity (age at first birth, litter size, birth interval), and movement patterns. For the duration of stay at a cluster, we combined the northern and southern regions and distinguished between pregnant and nonpregnant females. We used a one‐way ANOVA to compare the amount of time spent at a site by pregnant females and nonpregnant females between and within the southern and northern regions during a month when a birthing event was recorded. We then identified which movement variable (net displacement, duration of stay, and range size) were a function of variation in monthly birthing rate.

The month of conception and likely month of birth were calculated by aging all litters seen during monitoring and captures of both collared and noncollared adult pride females. Once the existence and location of denning sites with cubs had been verified, the approximate date and season in which conception occurred was identified by dating back three months. We measured whether a relationship between seasonality of annual reproductive patterns of lions and their prey existed as a function of regional monthly rainfall. Rainfall data were collected monthly from weather stations across the KNP for the duration of the study from January 2010 to December of 2012. Monthly rainfall allows for better‐observed detailed changes rather than annual rainfall.

The peak conception time of the southern and northern regions was compared using one‐way ANOVA and linear regression. The GLM's were used to determine whether there was a relationship between births and conceptions of lion in the southern and northern regions and (a) rainfall, (b) prey biomass, and (c) prey lambing and calving. Births and conceptions were calculated for each region separately and then compared. A Poisson regression on a binomial distribution was used for response variable: birth and conception, and explanatory variables: rainfall, prey calving, and lambing. In this distribution: 1 = successful birth or likely conception and 0 = no births or conception; 1 = peak prey births and 0 = low prey births; and 1 = wet season and 0 = dry season. The AIC*c*, AIC*∆i*, and the Akaike weight (*wi*) were then calculated.

To answer the first hypothesis, we used the movement pattern outcomes from the potential birth month calculations to identify reproductive activity. We calculated potential month when a birth occurred by the following metrics collected through GPS radio‐telemetry: (a) the duration of stay at a particular denning site by identifying GPS fix clusters; (b) core and total home range size during the potential birth month; (c) variation in daily net displacement (km) during the wet and dry season; and (d) and variation in daily net displacement (km) during potential birth month.

To address the second hypothesis, we calculated the association between the birth timing and ecological factors by measuring known birth and conception with (a) prey biomass, (b) lambing and calving of preferred prey, and (c) rainfall. We used verified cub births to identify whether there was an association between the occurrence of births and conception incidences and ecological factors.

To address the third hypothesis, we measured the fecundity of female lions in the KNP; (a) age at first birth, (b) litter size, and (c) birthing intervals. We compared the fecundity of lionesses in the KNP with other lion populations.

#### 2.1.1 Seasonality of reproduction

To address our first hypothesis, do lions show seasonality of reproduction, we analyzed movement patterns through net displacement, range size, and duration of stay at a site. Direct observations were conducted by ground proofing or camera traps to visually verify the presence of cubs and the collared female's reproductive state. To determine spatial use, the range size in a month of a birth was compared against the range size in the same month of a collared nonpregnant female in the same region. Once a litter was recorded, the variables of clusters (the number of consecutive fixes within a 200 m radius), daily net displacement, and range size for lions were grouped by region (southern and northern) and compared. The net displacements of collared pregnant and nonpregnant lionesses were compared using one‐way ANOVA (*p* < .05). Data of the collared pregnant and nonpregnant females were taken for the duration of the study between 2010 and 2012. Range size of core and total home ranges in the southern and northern region were compared using the one‐way ANOVA (*p* < .05). Data came from both collared females (*N* = 20) and adult female pride members that were verified as having birthed cubs (*N* = 12). The data used from the noncollared females were only for identifying birth and conception and not for range size or net displacement We used GLM's to identify which of the movement patterns, range size, daily net displacement, as explanatory variables were most indicative of the likelihood of a birth.

#### 2.1.2 Birth timing

To test the second hypothesis, of whether lion birth timing associate with ecological factors, we compared the ecological conditions in the form of monthly rainfall and prey biomass. We did not take into account landscape features or habitat quality because we used prey biomass as an indication of landscape and habitat quality. Prey biomass was associated with game counts and relative to lambing and calving of preferred prey. We used monthly rainfall because it indicated the variation between seasons in more detail than annual rainfall. Previous studies indicated that buffalo (Pienaar, 1969), wildebeest (Smuts, 1976), kudu (Smuts, Hanks, & Whyte, 1978), and zebra (Mason, 1990) have peak births during the wet season in southern Africa, usually in late October and January to March (Ogutu & Dublin, 2002). We included rainfall because previous research has indicated a strong relationship between lion behavior and prey activity and movement patterns (Tambling et al., 2010).

To investigate the relationship between lion birth timing and prey biomass, 15 prey species included in game counts were taken into account (Fairall, 1968; Kruger, Reilly, & Whyte, 2008). However, for the purpose of this study, only known preferred lion prey species were used to determine birth seasonality (Owen‐Smith & Mills, 2006; Radloff & Du Toit, 2004). These species included buffalo (*Syncerus caffer*), kudu (*Tragelaphus strepsiceros*), zebra (*Equus quagga burchellii*), and wildebeest (*Connochaetes taurinus*; Du Toit, 1995; Klingel, 1975; Pienaar, 1969; Skinner, Zyl, & Heerden, 1973). Game counts in the KNP have been recorded through annual censuses since 1970 between July and October; however, the method was changed in 1998 from total counts to sample surveys with an average of 22% coverage (Kruger et al., 2008). The change in methodology was found to be sufficiently precise in estimating herbivore populations (Kruger et al., 2008). The prey biomass was scaled to the total home range size of the pregnant female to adjust for prey availability. Essentially, the prey biomass data from the region where the female occurred were used. We then used GLM's to identify which of the explanatory variables, prey biomass and rainfall, were most contributing to the birth timing.

#### 2.1.3 Fecundity

To verify whether fecundity alters because of ecological factors, we analyzed the average age at first birth, birthing intervals, and litter size of collared females in the KNP.

Fecundity data were analyzed using one‐way ANOVA (*p* < .05) to identify whether there was variance in the fecundity of age at first birth, litter size, and birth intervals in the southern and northern regions. A Kruskal–Wallis test was used to determine whether there was a difference between the fecundity in the KNP when compared to other parks in east Africa (Serengeti National Park and Nairobi National Park), South Africa (Makalali Nature Reserve), and historical KNP data. We compared parks that were relatively of similar size and functioning under natural ecological and environmental conditions rather than smaller reserves that highly manage the lion populations. All statistical analyses were run in R version i386 2.15.1 Ink R Core Team (2013): (http://www.R-project.org/) [Accessed: 2013, 30 March] using the Rcmdr package (Fox, 2005).

## 3 RESULTS

Of the 20 collared females, 12 were recorded as having had cubs, 6 in the southern region and 6 in the northern region. From these collared females only, a total of 37 cubs were observed and recorded. When we included all pride females, both with and without collars, a total of 20 lionesses had cubs, nine lionesses in the southern region and 11 lionesses in the northern region. In total, 97 cubs were observed during the study period, 62 in the south and 35 in the north regions.

Of the 12 recorded births by collared females, 10 birth events were identified using net displacement and GPS clusters of the collared females to locate denning sites. Of these denning sites, eight were ground proofed by direct observations with litters belonging to the radio‐collared lionesses. For two lionesses, cubs were seen during captures in excess of five months after birth, which made it difficult to prove whether the collared female was the mother. For the remaining two lionesses, no ground proofing was possible. Of the radio‐collared lionesses with confirmed denning sites with litters, three in the southern and two in the northern region had synchronized births with other lionesses within the pride.

### 3.1 Seasonality of reproduction

**Figure 2:**
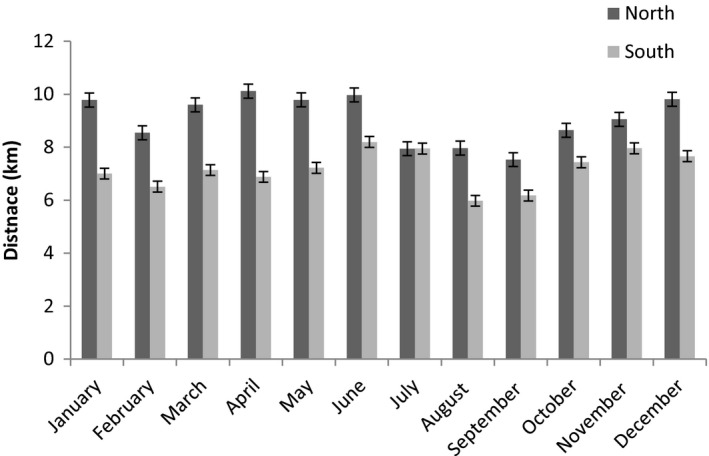
Average daily net displacement (km per day) per month of female lions in the Kruger National Park in the southern and northern regions between 2010 and 2012

**Figure 3:**
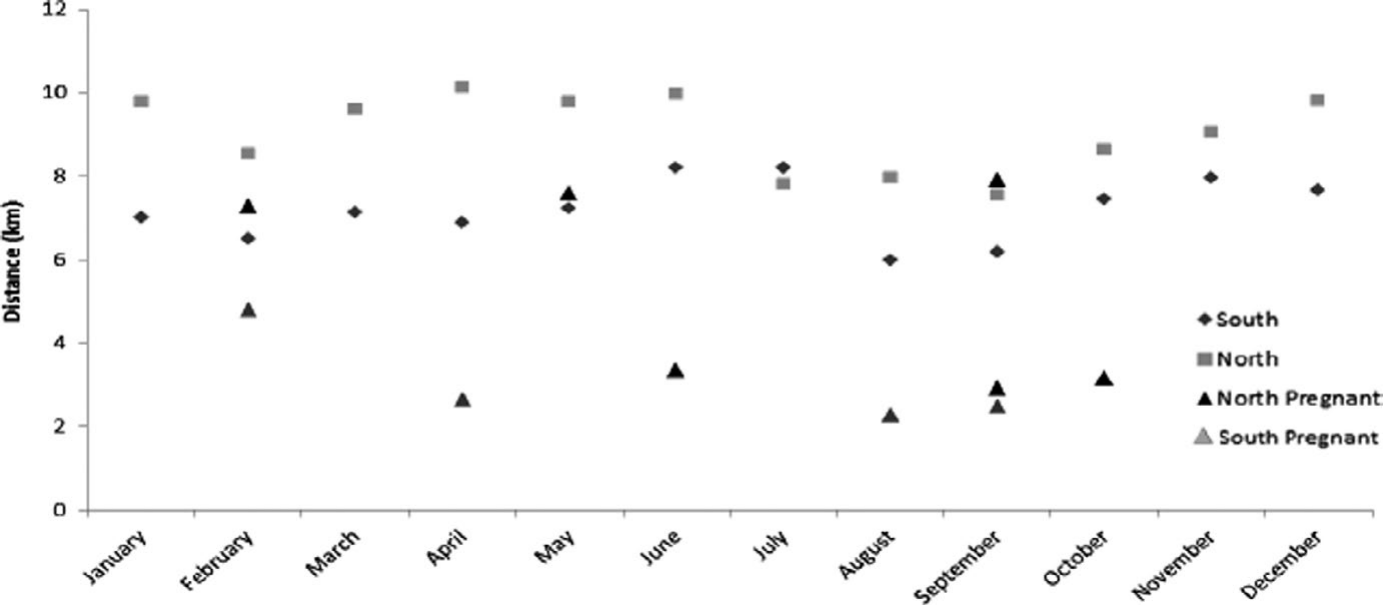
Average daily net displacement of nonpregnant and pregnant female lions during the month of parturition in the northern and southern regions of the Kruger National Park between 2010 and 2012

Lion prides in the southern region had shorter (mean = 7.2 km, *SE* = 0.9) daily net displacement than the prides in the northern region (mean = 9.1 km, *SE* = 1.1; Figure 2; *F*_5, 58_ = 6.53, *p* < .05). When compared, the pregnant females in the southern region had a shorter daily net displacement (mean = 4.3 km, *SE* = 1.3) than the pregnant females in the northern region (mean = 5.4 km, *SE* = 1.2); (*F*_2, 32_ = 0.42, *p* = .66). When the southern and northern pregnant females were compared with nonpregnant females during the birth month, the pregnant females had a shorter step length (distance between consecutive GPS fixes), than the nonpregnant females (*F*_2, 26_ = 3.87, *p* = .03). There was a 51%, reduction in net displacement in the northern (from 9.48 to 5.81 km) and southern (from 7.40 to 3.08 km) regions during the birth month (*SE* = 4.3, 95% CI; Figure 3).

Median total range size of the collared pregnant females differed between birth month and a seasonally similar month when the female was not pregnant for both core home range (*R* ^2^ = 0.608, *p* = .004) and total home range (*R* ^2^ = 0.655, *p* = .002; Table 1). During the birth month, collared pregnant females operated over a smaller core home range (mean = 0.2 km^2^, *SE* = 0.1) compared with the core range (mean 3.4 km^2^, *SE* = 1.7) of nonpregnant females (*F*_1, 12_ *= *24.55, *p* < .05). During the birth month, total home range of collared pregnant females (mean = 6.3 km^2^, *SE* = 3.4) was smaller compared with the total monthly home range (mean = 19.5 km^2^, *SE* = 12.2) of the nonpregnant females (*F*_1, 12_ = 7.55, *p* < .05). The best candidate GLM models to predict a successful birth incident and denning site comprised core range and the combination of core and total range (Table 1).

**Table 1:** Explanatory variables of identifying lion denning sites as a function of movement patterns in the Kruger National Park, South Africa

Model variables	*K*	AIC	AIC*c*	AIC*c∆i*	AIC*wi*
50% Core	1	14	14.22	0	0.74456
Core + Total	2	16.73	17.44	3.22	0.14885
90% Total	1	17.9	18.12	3.9	0.10593
Biomass	1	30	30.22	16	0.00025
SL Wet	1	30	30.22	16	0.00025
SL Dry	1	30.9	31.12	16.9	0.00016
BTb	1	33.36	33.59	19.36	4.65e^−5^

Note

Assessed through candidate general linear models (GLMs) with the number of parameters (*k*) using the Akaike information criterion (AIC). Included are the corrected (AIC*c*) for small sample size, the difference between the AIC candidate models and the smallest AIC recorded for the GLM (AIC*c∆I*), and the weight (*wi*) of the model.

Abbreviation: SL, step length.

During a birth month, the amount of time spent within a 200 m radius differed, but not significantly, between nonpregnant and pregnant females (*F*_4, 6_ = 0.731, *p* = .454). The average amount of time spent at a locality during a month when a birth event occurred was shorter for nonpregnant females (mean = 4.9 days, *SE* = 0.6) than that of pregnant females (mean = 19.7 days, *SE* = 1.7).

### 3.2 Birth timing

**Figure 4:**
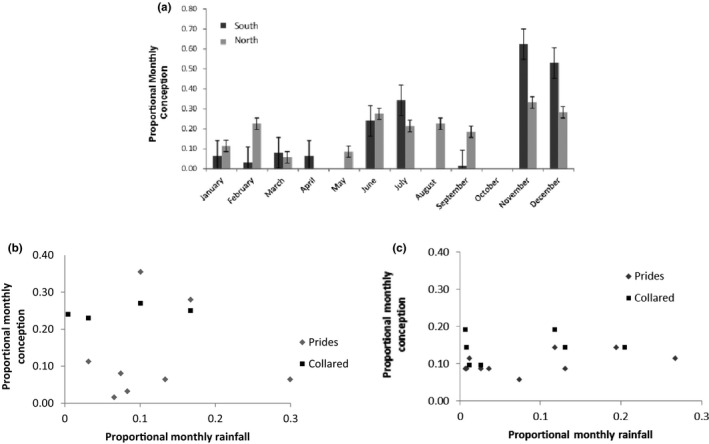
Patterns of conception in lions of the Kruger National Park with the monthly mean proportion of conception frequency and standard errors in southern and northern regions. (a) The association between proportional monthly rainfall and proportional monthly conception in the southern (b) and northern (c) regions

**Figure 5:**
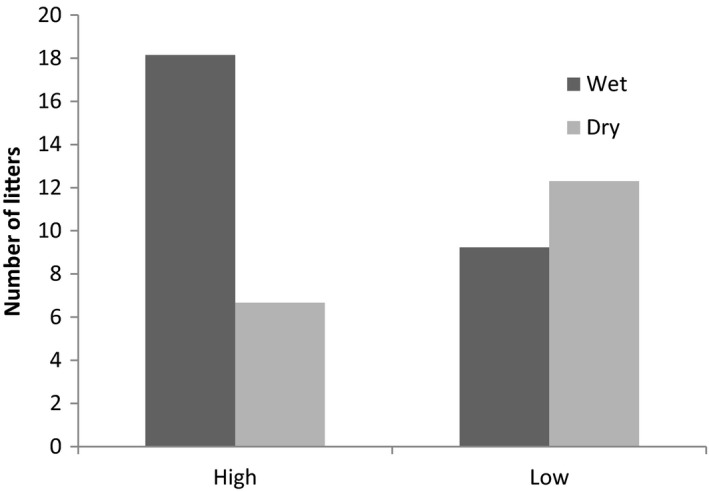
Seasonal distribution of lion births in the high prey biomass (southern) and low prey biomass (northern) regions of the Kruger National Park, South Africa

One‐way ANOVA suggested no relationship between rainfall and conception in the south (*F*_1, 7_ = 0.015, *p* > .05) and in the north (*F*_1, 8_ = 2.734, *p* > .05) regions (Figure 4). However, a weak relationship between rainfall and birth was present in the south (*F*_1, 2_ = 0.453, *p* > .05) and in the north (*F*_1, 5_ = 0.029, *p* > .05) region (Figure 5). Half of the birth events that were observed in the southern region took place in the wet season between November and February. We dated back three months from the birth month and estimated that 73% likelihood of conception in the early wet season between September and December and a 27% likelihood of conception in the dry season between June and August. In the northern region, 48% of the birth events took place in the wet season between September and February. We dated back three months from the birth month and estimated that 71% likelihood of conception took place in the wet season between October and December and 29% likelihood of conception in the dry season between March and August. The GLMs indicated a relationship between the months of lion conception and the births of preferred prey in both the southern and northern regions. The GLMs suggested little relationship of lion and preferred prey births in both regions (Table 2).

**Table 2:** Ecological explanatory variables of lion conception and births relative to rainfall preferred prey calving and lambing in the Kruger National Park, South Africa

Ecological Region	Model variables	*k*	AIC	AIC*c*	AIC*c∆i*	AIC*wi*
Birth	~ prey + rain					
Southern		2	19.32	20.03	2.91	0.14
Northern		2	21.05	21.75	4.64	0.06
Conception	~prey + rain					
Southern		2	18.64	19.35	2.23	0.20
Northern		2	16.41	17.12	0	0.60

Note

Assessed through candidate general linear models (GLMs) using the Akaike information criterion (AIC). Included are the corrected (AIC*c*) for small sample size, the difference between the AIC candidate models and the smallest AIC recorded for the GLM (AIC*c∆i*), and the weight (*wi*) of the model.

### 3.3 Fecundity

From the data collected in this study, there was no significant difference in the average age at first birth for lionesses in the southern (mean = 3.7 years, *SE* = 0.3) and northern (mean = 3.9 years, *SE* = 0.1) regions (*F*_2, 7_ = 0.3, *p* = .8). Nor was there a significant difference for litter size (south: mean = 2.6 cubs, *SE* = 0.3, and north: mean cubs = 2.7, *SE* = 0.3,); (*F*_2, 7_ = 0.24, *p* = .62). There was insufficient data from the pregnant females to compare birthing intervals (south *N* = 3; and north *N* = 0), which averaged 2.9 years (*SE* = 1.1) in the south.

Our findings for fecundity were compared with KNP historical data and data from other large reserves. No significant difference between the current data collected during this study, historical KNP data from Serengeti National Park, Nairobi National Park, and Makali Nature Reserve were found using the Kruskal–Wallis test (*X* ^2^_6_ = 6, *p* = .423). Nor was there a significant difference found in the fecundity of KNP between this study and 1978 (Smuts et al., 1978; *X* ^2^_3_ = 3, *p* = .416; Table 3).

**Table 3:** Reproductive variables of female lions in the Kruger National Park over 35 years: a comparison with prides in Tanzania, Kenya, and South Africa

Reproductive variables	Serengeti^a^	Makalali^b^	Nairobi^c^	Kruger^d^ (1978)	Kruger^e^ (2003)	Kruger^f^ south (2012)	Kruger^f^ north (2012)
Age 1st birth (years)	4.8	3.6	2.6	4.0	4.0	3.7 ± 0.015	3.9 ± 0.005
Litter size	2.5	2.9	3.0	3.1	3	2.7 ± 0.014	2.6 ± 0.016
Birth interval (months)	24.0	22.0	23.5	40	40	34.8 ± 0.06	—

Note

Data sources:

a Bertram (1975).

b Druce et al. (2004).

c Rudnai (1973).

d Smuts et al. (1978).

e Funston, Mills, Richardson, & van Jaarsveld (2003).

f This study.

## 4 DISCUSSION

Energetic and metabolic needs vary at different stages of reproduction, from conception, through gestation to birth (Woodroffe, Chapman, & Lemusana, 2009; Wu et al., 2004). Our findings indicated that core range size was a key indicator of a denning site and birth event. Although there was no correlation between rainfall and conception, there was a correlation between seasonality of lion conception and the birth peak of key preferred prey namely buffalo, kudu, zebra, and wildebeest. Prey biomass peaked during the early part of the wet season because of lambing and calving. In the southern and northern regions, fecundity data were largely the same and comparable to historical data in the KNP and in other large parks.

Pregnant female lions are elusive, and without the aid of remote detection, the identification of denning sites can be problematic (Woodroffe et al., 2009). The detection of altered movement patterns thus provides an indication of changes in behavior. Although not significant, duration of stay provided some support for indicating the location of denning sites. Note, however that the frequency of revisitation to denning sites may suggest the likelihood of a successful birth or use of key resources (Ruprecht, Ausband, Mitchell, Garton, & Zager, 2012). For example, during this study there were incidences of lions remaining at a locality for several days at a buffalo carcass. The ability to identify key features of denning sites is important to assist strategic management of landscapes for lion conservation (Abade, Macdonald, & Dickman, 2014). Understanding the landscape drivers of lion distribution assists with effective conservation efforts when developing or maintaining protected areas. This knowledge informs conservation managers about the type and quality of landscape features to include and monitor when expanding or establishing protected areas.

Although both pregnant and nonpregnant lionesses in the southern region had shorter daily net displacements than lionesses in the northern region, this was likely due to higher frequency of prey encounters, water distribution, and the general quality of the home ranges (De Boer et al., 2010; Loveridge et al., 2009; Ogutu & Dublin, 2004) in the southern region (Funston et al., 2003). Our results confirmed that pregnant females alter their range use at various phases of pregnancy. Reproduction is a physiological state that places the female body under stress because it requires higher amounts of energy input and output (Wu et al., 2004). Energy from food prepares the body for conception and sustains a pregnancy through the gestation period (Woodroffe et al., 2009). Accordingly, pregnant females need energy to sustain the pregnancy and, therefore, cannot expend it by walking great distances to find resources such as water, prey, and shelter (Du Toit, 1995; Wu et al., 2004). As expected, in our study the net displacement of all pregnant lionesses was substantially less (56%) than nonpregnant lionesses leading to significantly smaller core and total range of pregnant females compared with nonpregnant females in both the southern and northern regions during the birth month. This is consistent with the reduction in red wolf female and breeding pairs range size during birth and pup‐rearing months. Further investigation into the landscape and resource features of these core ranges would provide insight into the key causal determinants of core range selection and denning sites by female lions (Abade et al., 2014).

Our results revealed a correlation between the birthing peaks of preferred prey during the month of conception. Interestingly, while there was no correlation between conception and rainfall, there was evidence that peak conception occurred during the wet season when buffalo, wildebeest, kudu, and zebra have birth peaks—usually in late October and January to March (Mason, 1990; Pienaar, 1969; Smuts, 1976; Smuts et al., 1978). Coinciding conception with calving and lambing of preferred prey strategically allows for pregnant females to easily access resources needed to sustain a pregnancy (Holekamp et al., 1999; Ogutu & Dublin, 2002). The wet season conception peaks identified in our study most likely indicate lionesses accessing food and water resources to sustain body conditions that facilitated conception. Lion cubs start eating meat at three months of age and are completely weaned by six months. Prior to birth and for the first three months after birth, a lioness is often accompanied by another female that assists with cub rearing and hunting. This companionship alleviates energy output that would otherwise be associated with cub rearing (Packer et al., 2005). It is anticipated that once the cubs start eating meat exclusively at six months and onwards the energy output of a female increases. However, this would be balanced by cooperative group hunting.

In Kenya, spotted hyena births coincide with response to season variation and resource availability (Holekamp et al., 1999), although spotted hyenas can breed year‐round. Likewise, lions in the Maasai Mara in Kenya displayed birth peaks during the wet season between March and June, coinciding with the increase in migratory prey (Ogutu & Dublin, 2002). In North Carolina, red wolves prey predominantly on white‐tailed deer (*Odocoileus virginianus*), especially the fawns, during pup‐rearing months (Dellinger et al., 2011). Similarly, wild dogs in northern Kenya survived on small prey that a single wild dog could capture when pregnant or rearing small pups (Woodroffe et al., 2009).

Our third hypothesis, fecundity alters as a result of ecological factors, was not supported by our results. Firstly, litter size, age at first reproduction, and birthing interval were not significantly different between the resource‐rich southern region and poorer resourced northern regions. Secondly, when compared with historical data (Smuts, 1976; Smuts et al., 1978), and data from lions in the Nairobi National Park (Rudnai, 1973) and Makalali Nature Reserve (Druce et al., 2004) all representing differing resource conditions, the fecundity of lions in our study fell within the same range. Accordingly, our study suggests that ecological conditions are important, but they are not drivers of the fecundity of lions in the KNP. There may be other factors unaccounted for that drive fecundity. This study, however, identified ecological mechanisms that may influence conception.

Using movement patterns as behavioral indicators, we were able to detect the denning sites remotely and identify reproductive activities of lions. In existing protected areas, conservation practitioners can be more selective in their management of denning sites to avoid burning and minimize disturbance during denning. In the event of poaching activity on a reserve, conservation mangers will be better suited to direct law enforcement to the denning sites. Although this study did not focus or take into account poaching of wild lions, the lessons learned could be applicable to addressing such incidences. Kruger National Park has not been identified as a source of lion poaching. Lions that have been killed outside the park are ad hoc animals that have left the park and entered neighboring communal property. Throughout Africa, retaliation killing for livestock lose is one of the primary human–wildlife interactions leading to death and population declines of lions (Tumenta et al., 2010). Although the trade in lion bones has put a spotlight on lion parts for trade, this is most evident in captive‐bred lions more so than wild lions (Department of Environmental Affairs South Africa, 2018). The ability for conservation managers to detect births and denning activities of lionesses allows for better preparedness in the event of a poaching incident.

This study provided insight into areas of study not previously questioned such as the importance of conception rather than just the birthing. Population dynamics could consequently be a function of poor conditions during conception and gestation. Similar to the importance of understanding the role of landscape features and habitat quality in lion denning site selection, so is understanding the triggers and drivers of conception. This study suggests that space use in the form of landscape features and habitat quality have an impact on reproduction. Although this study was conducted in an area with known bTB disease prevalence, disease did not appear to be a significant driver.

It is important to note that this study did not explore social cues of synchronized births of pride members or the effect of male takeovers. To strengthen our analysis, social cues in the form of male takeovers, estrus synchrony, and crèche formations could be incorporated. Nevertheless, ecological and environmental cues that influenced the conception period were identified, particularly regarding the influence of births of preferred prey. Identifying the resources and landscape features associated with denning sites is an important next step to explore the effect of seasonality and elucidate denning site characteristics.

## CONFLICT OF INTEREST

None declared.

## AUTHOR CONTRIBUTIONS

All authors contributed equally to this work including the conception, data analysis, and manuscript synthesis.

## Data Availability

Data will be stored on the SANParks Data Repository and made accessible from http://dataknp.sanparks.org/sanparks/metacat/judithk.112037.1/sanparks, on request from SANParks or the corresponding author.
